# Time-course of post-activation performance enhancement (PAPE) following multi-dimensional elastic band vs. heavy resistance exercise

**DOI:** 10.3389/fphys.2026.1804907

**Published:** 2026-05-13

**Authors:** Yiming Liu, Xinmao Zou, Dong Lu, Ning Xu, Mengqi Chen, Liming Zhu

**Affiliations:** 1 Guangzhou Huali College, Guangzhou, China; 2 Guangdong Polytechnic Normal University, Guangzhou, China; 3 Guangdong Vocational Institute of Sport, Guangzhou, China; 4 Guangzhou Polytechnic of Sports, Guangzhou, China

**Keywords:** PAPE, variable resistance, vertical jump, peak power, warm-up, time-course

## Abstract

**Purpose:**

This study compared the acute time-course effects of a Multi-dimensional Elastic Band (MEB) activation protocol versus a traditional Heavy Barbell Squat (HBS) protocol on lower limb explosive performance, aiming to identify optimal recovery windows.

**Methods:**

Sixteen trained male athletes (age: 19 ± 0.9 yrs) participated in a randomized crossover study consisting of three sessions: Control, HBS (4 reps at 90% 1RM), and MEB (continuous vertical jumps against elastic resistance). Countermovement jump (CMJ) height and Peak Power Output (PPO) were measured at 3, 6, 9, 12, 15, and 18 minutes post-activation.

**Results:**

A significant condition × time interaction was observed for CMJ height (p = 0.039), which was primarily driven by the divergent time-course trends between conditions in the early (3-6 min) and late (12-18 min) recovery phases. *Post-hoc* pairwise comparisons did not reach statistical significance (p > 0.05), while effect size analysis (HBS and MEB vs. Control at each time point) indicated practical improvements. The MEB protocol induced a rapid potentiation response, demonstrating moderate effect sizes in CMJ height and PPO as early as 3 minutes post-activation. In contrast, the HBS protocol exhibited delayed potentiation, with improvements appearing only after 9 minutes, likely due to residual fatigue.

**Conclusion:**

Multi-dimensional elastic resistance is a time-efficient alternative for inducing Post-activation Performance Enhancement (PAPE), offering a rapid onset of practical potentiation (within 3 min) based on effect size analysis. These findings suggest MEB is particularly suitable for pre-competition warm-ups where recovery time is severely constrained.

## Highlights

- Multi-dimensional elastic band (MEB) activation elicits a rapid onset of PAPE (within 3 min).
- Traditional heavy resistance loading requires >9 min recovery due to fatigue masking effects.
- MEB offers a wider optimal window (3–15 min) compared to heavy resistance.
- Elastic resistance is recommended for pre-competition warm-ups where time is constrained.

## 1 Introduction

Explosive power is a determinant factor for success in many competitive sports, particularly those involving sprinting, jumping, and rapid changes of direction. To optimize these capabilities, athletes routinely perform specific warm-up routines designed to mobilize the neuromuscular system and enhance physiological readiness (McGowan et al., 2015; Silva et al., 2018). Beyond general warm-ups, the phenomenon known as Post-activation Performance Enhancement (PAPE) has garnered significant attention as a strategy to acutely improve voluntary muscular performance following a conditioning activity (CA) (Blazevich and Babault, 2019; Olsen et al., 2025). Distinct from the short-lived electrical potentiation of classical post-activation potentiation (PAP), PAPE relies on a net balance where the potentiation effect outweighs the coexisting fatigue induced by the CA (Hodgson et al., 2005; Till and Cooke, 2009; Karabel and Makarac, 2025).

Traditionally, high-intensity resistance exercises, such as heavy back squats (>85% 1RM), are the most widely used and mechanistically well-characterized conditioning activity for inducing PAPE in both research and applied settings (Seitz et al., 2014; Bauer et al., 2019) . A large body of literature has confirmed that this loading paradigm can effectively induce myosin light chain phosphorylation and recruit high-threshold fast-twitch motor units, which are the core neurophysiological mechanisms underlying PAPE (Hamada et al., 2003; Stull et al., 2011) . Due to its robust potentiation effect, heavy back squat has become the most commonly used protocol in PAPE mechanistic research, forming the basis for understanding the fitness-fatigue balance model of PAPE (Chen et al., 2023). However, the practical application of heavy resistance protocols presents several challenges. High-intensity loads often induce significant neuromuscular fatigue and while some studies have reported potentiation effects within short recovery windows (Mola et al., 2014), literature indicates that a prolonged recovery interval (typically >8 minutes) is often required before net performance benefits manifest (Gouvêa et al., 2013; Sharma et al., 2018), as fatigue must dissipate to reveal the underlying potentiation (Kilduff et al., 2007; Crewther et al., 2011). This prolonged recovery window can be logistically difficult to manage during competition warm-ups, where timing is often constrained.

To address these limitations, practitioners have sought alternative PAPE induction methods that offer better fatigue management, including variable resistance training (VRT), bodyweight ballistic exercises, inertial training, and isometric conditioning activities (Pereira et al., 2022; Dos Santos Silva et al., 2023; Fu et al., 2023; Terbalyan et al., 2025). Among these alternative methods, VRT, specifically using elastic bands, has emerged as a particularly promising option for pre-competition warm-ups. Unlike constant gravitational loads, VRT provides accommodating resistance where the load increases throughout the concentric range of motion, matching the human strength curve more effectively and reducing mechanical stress at the weakest joint angles (Mina et al., 2014; Joy et al., 2016). Multi-dimensional elastic resistance platforms (e.g., VertiMax) provide consistent tension throughout the entire jump movement cycle (eccentric, amortization, and concentric phases), promote inter-muscular coordination patterns that closely mimic sport-specific vertical jump and sprint movements, and may induce neuromuscular potentiation with lower metabolic fatigue compared to constant heavy loads (Rhea et al., 2008; Cardozo et al., 2024). Despite the theoretical advantages of such functional activation methods, recent systematic reviews have highlighted that limited research has directly compared the time-course effects of multi-dimensional elastic resistance versus traditional heavy constant loading on lower limb explosive power (Chen et al., 2023; Zhou et al., 2026a; Zhou et al., 2026b) and the optimal recovery window for elastic resistance-induced PAPE remains poorly characterized (Kilduff et al., 2008; Rhea et al., 2008).

Therefore, the purpose of this study was to compare the acute time-course effects of a Multi-dimensional Elastic Band (MEB) protocol versus a Heavy Barbell Squat (HBS) protocol on lower limb explosive performance. We hypothesized that: (1) The MEB protocol would induce a PAPE effect with a shorter recovery latency, operationalized as the first time point post-activation where a medium effect size in CMJ performance vs. Control is observed; (2) The MEB protocol would result in a longer potentiation window, operationalized as the total duration post-activation where practical performance improvements vs. Control are maintained, compared to the HBS protocol.

## 2 Methods

### 2.1 Participants

Sixteen male collegiate athletes from the School of Physical Education at Guangzhou Sport University volunteered to participate in this study. The participants’ physical characteristics were as follows (mean ± SD): age 19.0 ± 0.9 years, height 178.5 ± 5.2 cm, body mass 70.4 ± 6.8 kg, and 1RM back squat 118.9 ± 6.3 kg. Inclusion criteria were: (1) at least 2 years of resistance training experience; (2) familiarity with the back squat technique; and (3) no history of lower limb musculoskeletal injuries in the past 6 months. Participants were instructed to refrain from strenuous exercise, caffeine, and alcohol for 24 hours prior to each testing session. All experimental procedures were conducted in accordance with the Declaration of Helsinki, and the study protocol was reviewed and approved by the Ethics Committee of the Third Affiliated Hospital of Guangzhou Medical University (Approval No: 2021-046). All participants were fully informed of the experimental procedures, potential risks, and their right to withdraw at any time without penalty and provided written informed consent prior to the start of the study.

### 2.2 Experimental design

A fixed-sequence single-group three-period crossover design was employed to compare the effects of three different warm-up protocols on lower limb explosive performance and muscle activation. This design was selected to eliminate inter-individual variability, which is a major confounding factor in exercise performance research. Participants visited the laboratory on four separate occasions. The first session was for familiarization and baseline 1RM back squat testing. The subsequent three sessions involved the experimental conditions: (1) Control (Standard Warm-up only), (2) Heavy Barbell Squat (HBS) activation, and (3) Multi-dimensional Elastic Band (MEB) activation. A washout period of at least one week was implemented between consecutive experimental sessions to eliminate any potential carry-over effects of the previous intervention. All testing was conducted at the same time of day (14:00–17:00) for all participants to control for circadian rhythm variations in physical performance.

### 2.3 Procedures

1RM Testing: One week prior to the experimental trials, participants’ 1RM back squat was determined following the National Strength and Conditioning Association (NSCA) guidelines (Baechle and Earle, 2008). The testing protocol began with a general warm-up, followed by specific warm-up sets: 10 repetitions at 50% of estimated 1RM, followed by 5 repetitions at 70%. The load was then gradually increased (by 5-10%) until the participant could not complete a repetition with proper technique. A successful repetition was defined as the participant descending until the tops of the thighs were at least parallel to the floor and returning to the starting position. Rest periods of 3-5 minutes were provided between maximal attempts to ensure ATP-PCr replenishment.

Standard Warm-up (Control): This protocol served as the foundation for the intervention groups. It consisted of 5 minutes of jogging at a moderate pace, followed by 5 minutes of dynamic stretching (e.g., high knees, butt kicks, lunges).

Heavy Barbell Squat (HBS) Protocol: Following the standard warm-up, participants performed a high-intensity activation set designed to induce Post-activation Potentiation (PAP). This involved 4 repetitions of the back squat at 90% of their predetermined 1RM. This intensity was selected based on previous literature suggesting that heavy loads (>85% 1RM) are optimal for recruiting high-threshold motor units. A 3-minute passive rest interval was allowed between the standard warm-up and the activation set to allow for initial cardiovascular recovery. Safety spotters were present during all heavy lifts.

Multi-dimensional Elastic Band (MEB) Protocol: Following the standard warm-up, participants performed a specific activation routine using a VertiMax platform (VertiMax V8, Tampa, FL, USA) a device previously validated for developing lower-limb power (Rhea et al., 2008; McClenton et al., 2008). The protocol involved continuous vertical jumps with arm swings against elastic resistance. The intensity was monitored via heart rate (Polar H10), maintained between 170–190 bpm to ensure high-intensity output, corresponding to an approximate resistance load of 37.5 kg derived from the elastic tension.

### 2.4 Data collection

Jumping Performance: Countermovement Jumps (CMJ) were assessed to evaluate vertical explosive power, a valid and reliable measure of lower limb explosive performance in athletic populations (Makaracı et al., 2021). A Smart Jump contact mat system (Fusion Sport, Coopers Plains, Australia) was utilized to measure flight time (ms) with millisecond precision. Participants were instructed to stand with hands on hips to isolate lower-limb power and minimize arm-swing contribution. Upon the “go” command, they performed a rapid downward movement followed immediately by a maximal vertical jump. Measurements were taken at baseline (pre-activation) to establish a reference value, and subsequently at 3, 6, 9, 12, 15, and 18 minutes post-activation. This frequent sampling allowed for the detailed mapping of the PAPE time-course.

Kinetic Variables: To provide mechanistic insight, Peak Power Output (PPO) and Leg Stiffness (K_leg_) were derived. PPO was calculated using the validated Sayers equation based on jump height and body mass. Leg Stiffness was estimated using flight time and contact time data, serving as an indicator of neuromuscular efficiency and elastic energy utilization (Sayers et al., 1999).

### 2.5 Statistical analysis

Data are presented as mean ± standard deviation (SD). Prior to analysis, the normality of the data distribution was verified using the Shapiro-Wilk test. A two-way repeated measures analysis of variance (ANOVA) [3 Conditions (Control, HBS, MEB) × 6 Time Points] was used to determine the main effects and interaction effects of the protocols on performance (CMJ Height) and kinetic variables (PPO, Stiffness). Statistical power was calculated using G*Power software (Faul et al., 2007). Effect sizes (Partial Eta Squared) were interpreted according to Cohen’s guidelines (Cohen, 1988). Effect sizes (Cohen’s d) were calculated for HBS and MEB conditions versus the Control condition at each time point to assess the practical significance of performance changes, with thresholds of 0.2 for small effects, 0.5 for medium effects, and 0.8 for large effects. In addition, within-subject repeated-measures effect sizes (partial eta squared, η²G) were reported for the ANOVA results to quantify the magnitude of interaction and main effects. If significant interaction effects were found, Bonferroni-adjusted *post-hoc* tests were performed to identify the specific locations of pairwise differences between conditions at each time point. The significance level was set at p < 0.05. All statistical analyses were performed using SPSS software version 26.0 (IBM Corp., Armonk, NY, USA).

## 3 Results

### 3.1 Baseline characteristics

There were no significant differences between the three conditions (Control, HBS, MEB) at baseline for any of the performance (CMJ height) (p > 0.05), indicating that the participants were in a similar physical state prior to each intervention session (**Table 1**).

**Table 1:** Descriptive characteristics of the participants (N = 16).

Variable	Mean ± SD
Age (years)	19.0 ± 0.9
Height (cm)	178.5 ± 5.2
Body Mass (kg)	70.4 ± 6.8
Body Mass Index (kg/m²)	22.1 ± 1.5
1RM Back Squat (kg)	118.9 ± 6.3
Relative Strength (1RM/BM)	1.69 ± 0.12

Values are presented as Mean ± SD. 1RM, One-repetition maximum; BM, Body Mass.

### 3.2 Jumping performance

#### 3.2.1 Countermovement jump height

A two-way repeated-measures ANOVA revealed no significant main effect of warm-up condition (F_2, 22_ = 0.79, p = 0.465, η^2G^​ = 0.011) or time (F_2.85, 31.35_ = 2.356, p = 0.093, η^2G^​ = 0.023). However, a significant condition × time interaction effect was observed (F_10, 110_ = 2.01, p = 0.039, η^2G^ = 0.019), indicating that the time-course trajectories of jump performance differed between the three warm-up protocols. *Post-hoc* pairwise comparisons with Bonferroni correction revealed no statistically significant differences between HBS or MEB and the control condition at any individual time point (all adjusted p > 0.05).To explore the practical significance of the observed trends, we conducted effect size analysis (Cohen’s d) for each condition versus the control, which provides complementary information to the inferential ANOVA results.

**Figure 1:**
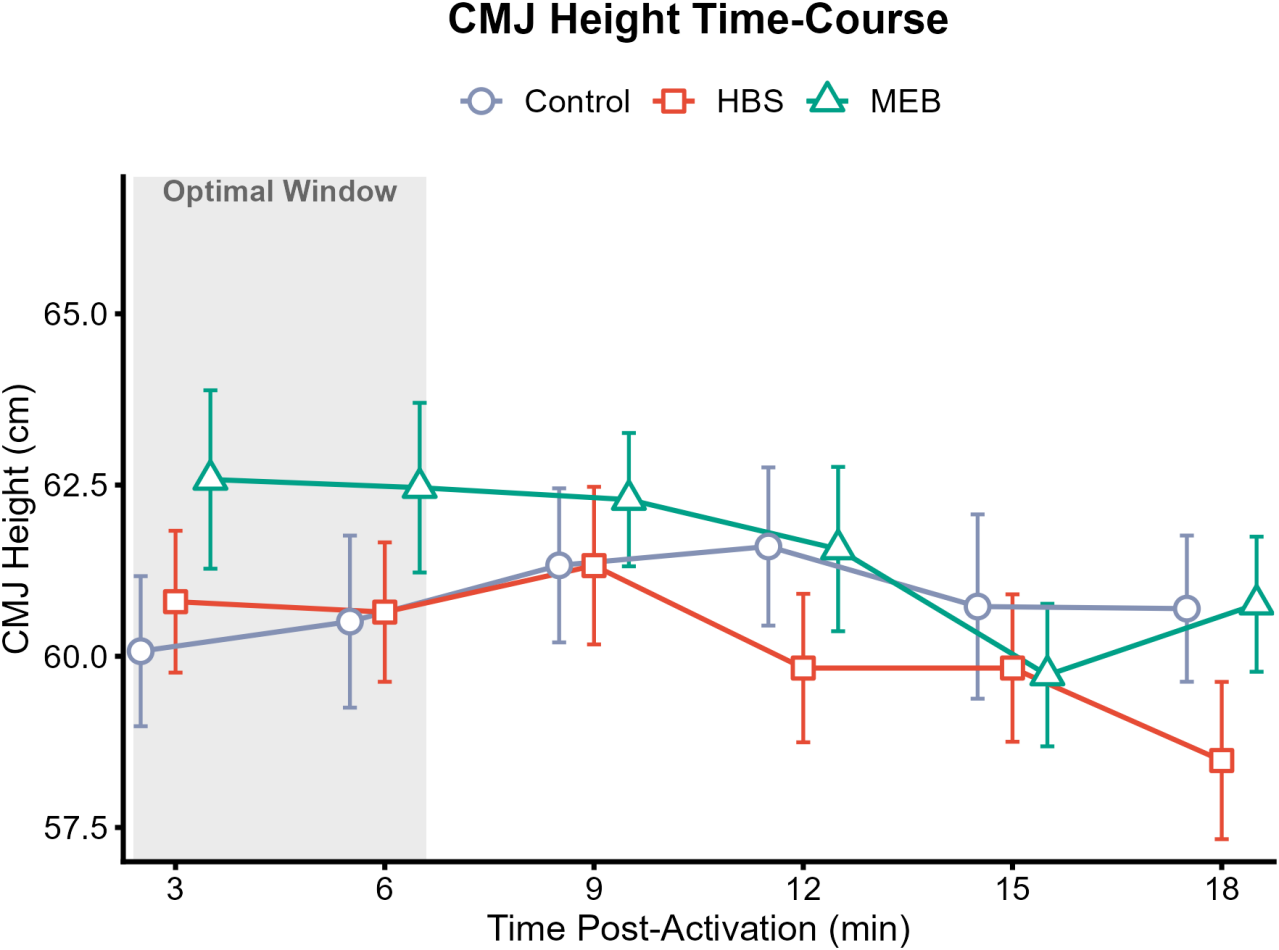
Time-course changes in Countermovement Jump (CMJ) Height across time points for Control, HBS, and MEB conditions.

As illustrated in **Figure 1**, the MEB condition showed a slight trend of earlier improvement compared to the HBS condition. The magnitude of differences between conditions was modest, with mean differences of approximately 1–2 cm observed at all time points, and error bars showed substantial overlap between groups throughout the testing period.

The peak CMJ height in the MEB group was observed at the 3-minute mark, falling within the 3–6 min post-activation window (**Figure 1**). In contrast, the HBS condition showed no improvement at 3 minutes post-activation, with peak values reached at the 9-minute interval. The MEB condition had marginally higher mean values than HBS during the early recovery phase (3 min and 6 min).

**Figure 2:**
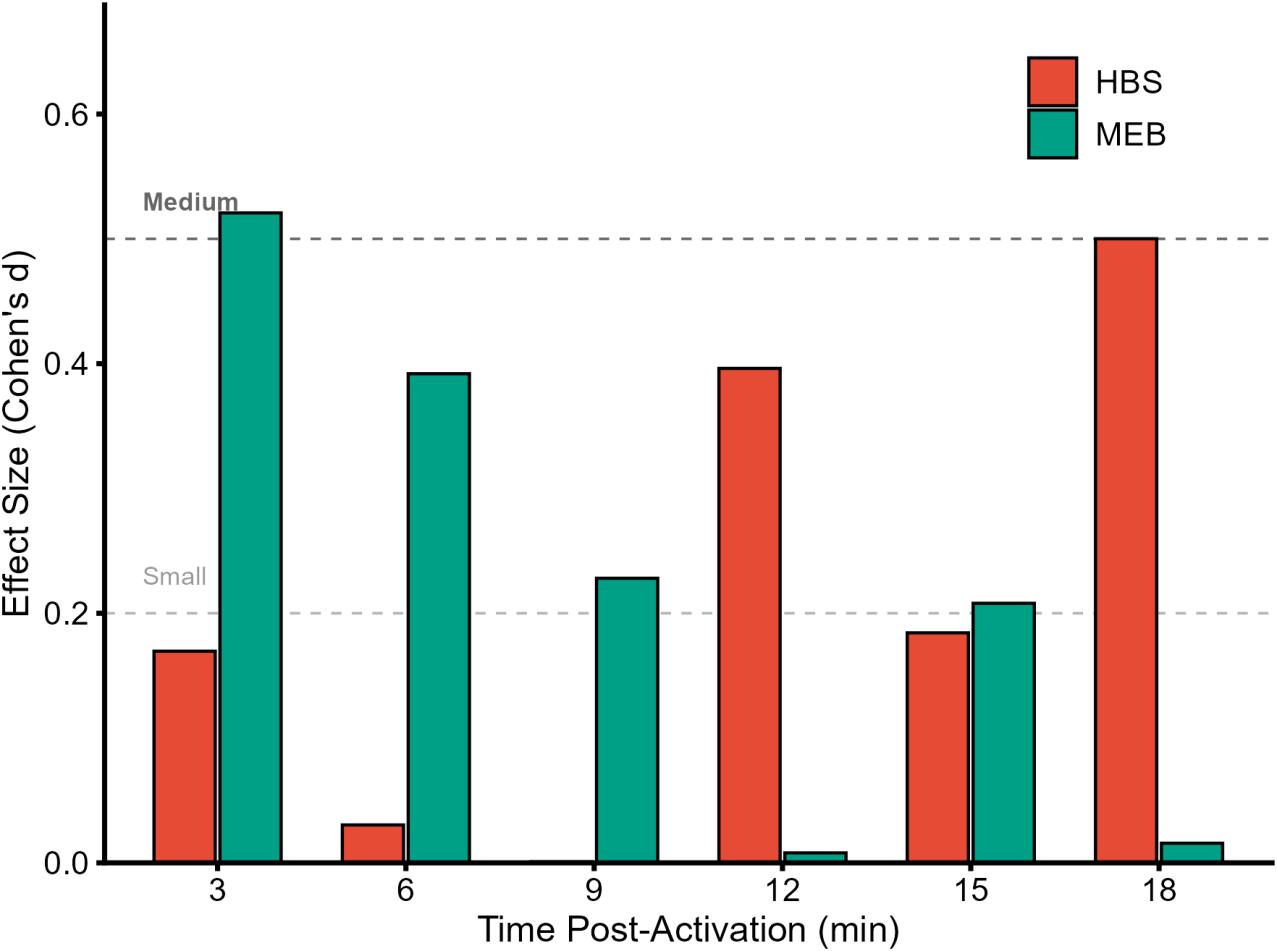
Effect sizes (Cohen’s d) for CMJ height comparing HBS and MEB against Control at each time point. Dashed lines represent thresholds for small (0.2) and medium (0.5) effects.

To quantify the practical significance of these trends, paired within-subject Cohen’s d effect sizes were calculated (**Figure 2**). The MEB protocol elicited a moderate effect size (*d* = 0.52) at 3 minutes post-activation and approached a moderate effect at 6 minutes (*d* = 0.39). In contrast, the HBS condition showed only trivial to small effect sizes during this early window, with its largest effect observed at 12 minutes post-activation (*d* = 0.40).

### 3.3 Kinetic variables

#### 3.3.1 Peak power output

Consistent with the CMJ height results, no significant main effect of warm-up condition (F_1.18, 12.98_ = 0.326, p = 0.614, η^2G^ = 0.011) or time (F_2.85, 31.35_ = 2.356, p = 0.093, η^2G^ = 0.022; Greenhouse-Geisser corrected) was observed for peak power output. A significant condition × time interaction effect was also detected (F_10, 110_ = 2.01, p = 0.039, η^2G^ = 0.018). *Post-hoc* pairwise comparisons with Bonferroni correction showed no statistically significant differences between conditions at any specific time point (all adjusted p > 0.05).

**Figure 3:**
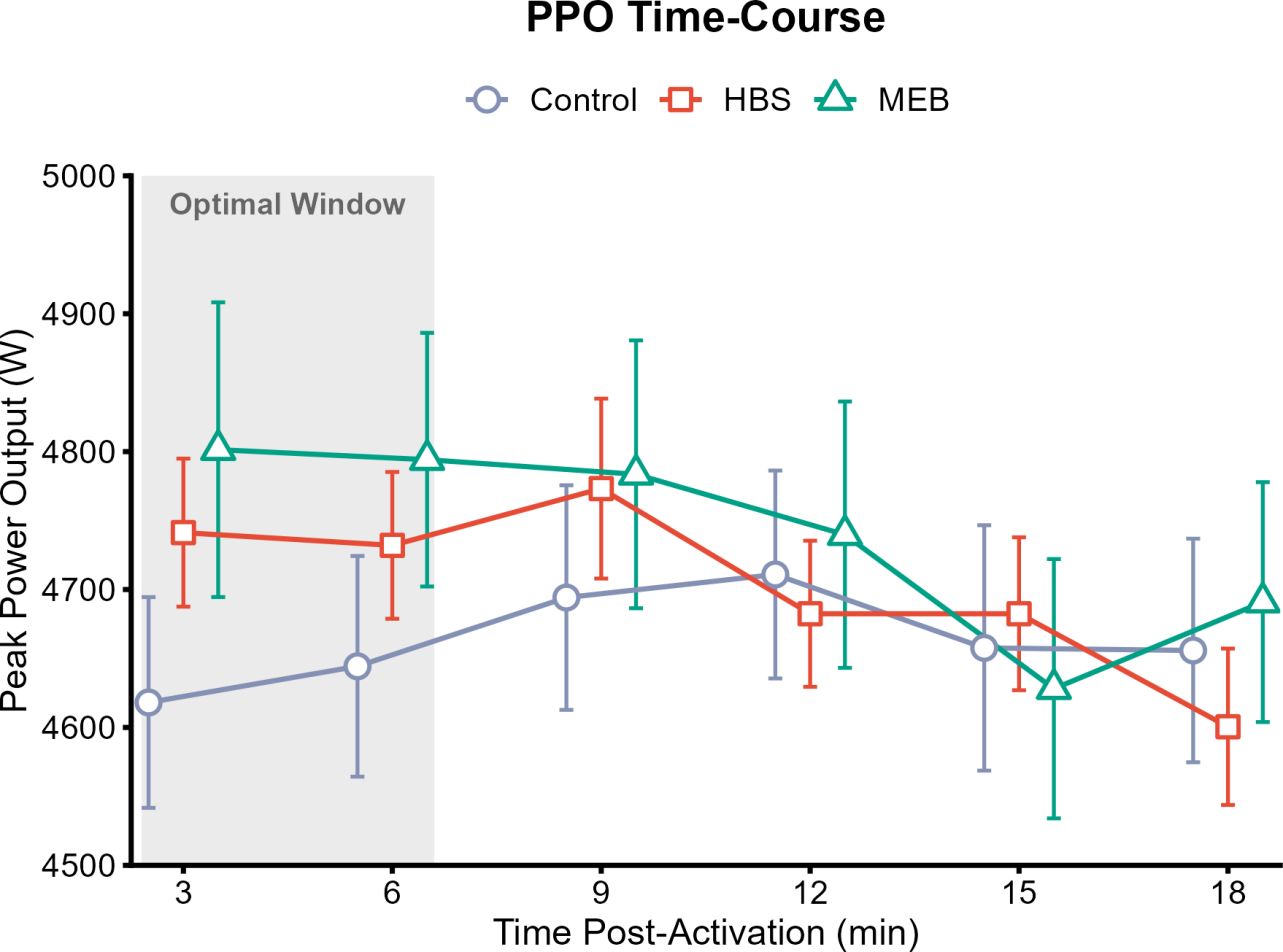
Time-course changes in Peak Power Output (PPO).

Similar to jump height, the MEB group showed a slight trend of higher power output during the early recovery phase compared to the control and HBS groups, with mean differences of approximately 50–100 W. The HBS group reached its peak PPO later, at 9 minutes post-activation (**Figure 3**).

**Figure 4:**
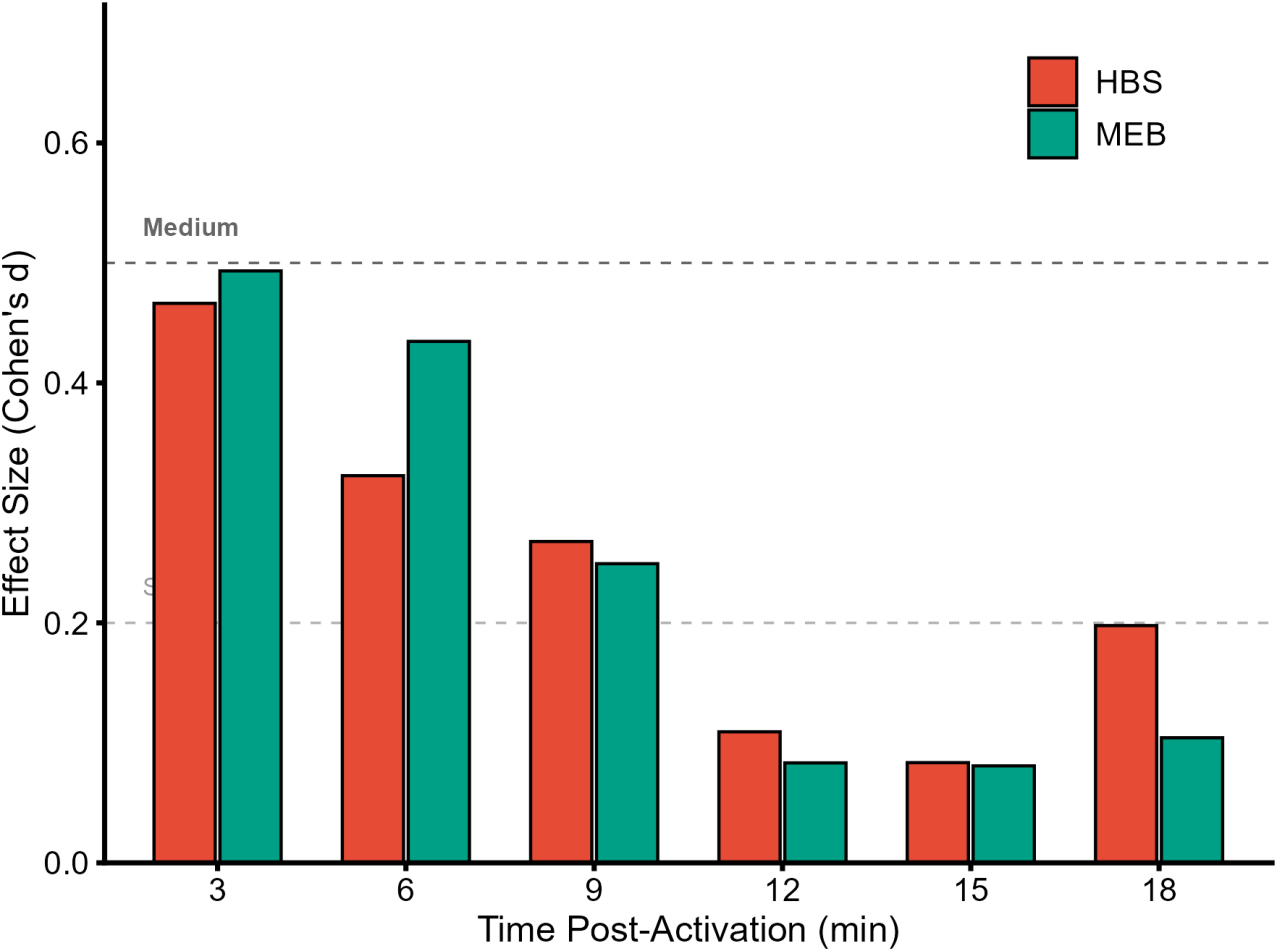
Effect sizes (Cohen’s d) for Peak Power Output (PPO) comparing HBS and MEB against Control.

Practical benefit analysis using paired within-subject Cohen’s d effect sizes is presented in **Figure 4**. The MEB protocol demonstrated an effect size approaching moderate magnitude (d = 0.48) immediately post-activation (3 min). In contrast, HBS showed smaller effect sizes that peaked later at 9 minutes (d = 0.27).

#### 3.3.2 Leg stiffness and flight time

Analysis of Leg Stiffness revealed no significant main effects or interactions (p > 0.05), indicating that both activation protocols maintained stable stiffness levels throughout the recovery period (**Table 2**).

**Table 2:** Descriptive statistics (Mean ± SD) for Jumping Performance (CMJ Height) and Kinetic Variables (Peak Power, Flight Time, Leg Stiffness) at baseline and post-activation time points.n ± SD).

Variable / Time	Control	HBS	MEB
CMJ height (cm)			
3 min	60.1 ± 4.4	60.8 ± 4.1	62.6 ± 5.2
6 min	60.5 ± 5.0	60.6 ± 4.1	62.5 ± 5.0
9 min	61.3 ± 4.5	61.3 ± 4.6	62.3 ± 3.9
12min	61.6 ± 4.6	59.8 ± 4.3	61.6 ± 4.8
15min	60.7 ± 5.4	59.8 ± 4.3	59.7 ± 4.2
18min	60.7 ± 4.3	58.5 ± 4.6	60.8 ± 3.9
Peak power output (W)			
3 min	4618.09±305.66	4741.25±214.70	4801.39±427.43
6 min	4644.32±320.05	4732.02±212.88	4794.16±367.85
9 min	4694.17±325.72	4773.20±260.91	4783.54±388.48
12min	4710.89±301.50	4682.43±211.95	4739.75±386.28
15min	4657.67±355.86	4682.43±221.76	4628.08±376.10
18min	4655.80±324.27	4600.47±226.80	4690.87±347.94
Flight time (ms)			
3 min	699.50±25.24	703.75±23.81	713.81±29.44
6 min	701.88±29.10	702.88±23.57	713.19±27.96
9 min	706.75±25.80	706.69±26.94	712.38±22.25
12min	708.31±26.51	698.06±25.29	708.06±27.48
15min	703.06±31.31	698.06±25.32	697.50±24.23
18min	703.12±24.89	690.06±27.24	703.56±22.90
Leg stiffness (kN/m)			
3 min	0.01±0.01	0.04±0.09	0.02±0.05
6 min	0.02±0.02	0.03±0.04	0.02±0.04
9 min	0.01±0.02	0.01±0.01	0.02±0.02
12min	0.02±0.02	0.02±0.01	0.02±0.02
15min	0.02±0.03	0.01±0.01	0.03±0.03
18min	0.02±0.02	0.01±0.01	0.02±0.03

For Flight Time, a significant condition × time interaction was observed (F_7.93, 178.42_ = 2.09, p = 0.040, η^2G^ = 0.018). Similar to CMJ height, pairwise comparisons did not reach statistical significance (p > 0.05), but the MEB group exhibited a trend of longer flight times during the early recovery phase (3–12 min) compared to the Control group.

## 4 Discussion

The primary purpose of this study was to compare the acute time-course effects of a Multi-dimensional Elastic Band (MEB) activation protocol versus a traditional Heavy Barbell Squat (HBS) protocol on lower limb explosive performance. The most significant finding of the present study was that the MEB protocol induced a PAPE effect with an earlier onset trend (observed at 3 min) and a longer duration of practical performance improvement (up to 15 min, based on effect size thresholds) compared to the HBS protocol. Specifically, the MEB condition showed trends of enhanced vertical (CMJ) jump performance during the early recovery phase (3–6 min), whereas the HBS condition exhibited a delayed potentiation window, with practical improvements appearing only after 9 minutes of recovery. These results support our hypothesis that multi-dimensional elastic resistance is a more time-efficient strategy for inducing PAPE than heavy dynamic constant external resistance. While HBS has long been considered a primary method for potentiation, our data suggests that its utility is strictly time-dependent, whereas MEB offers a more versatile “rapid-response” mechanism for acute performance enhancement.

The delayed potentiation observed in the HBS group (90% 1RM) is primarily consistent with the theoretical framework of the classic fitness-fatigue theoretical framework (Blazevich and Babault, 2019; Rassier and Macintosh, 2000), This model posits a dualistic response to conditioning activities, suggesting that the net effect on voluntary performance is determined by the dynamic algebraic balance between the induced physiological potentiation and the simultaneous coexisting neuromuscular fatigue (Hodgson et al., 2005). While heavy resistance training effectively recruits high-threshold motor units—a prerequisite for potentiation—it concurrently induces substantial levels of neuromuscular fatigue. This fatigue can effectively “mask” the potentiating effects during the immediate post-activation period, delaying the window of performance enhancement (Kilduff et al., 2007). In the specific context of our study, the HBS group exhibited either no improvement or a slight decrement in performance at the 3-minute mark, strongly suggesting that the fatigue component remained the dominant factor at this early stage of recovery.

However, fatigue alone cannot fully account for the observed 9-minute delay in peak potentiation following HBS. Three additional interrelated physiological and mechanical factors likely contribute to this temporal profile. First, myosin light chain phosphorylation itself follows a characteristic bell-shaped time course, with peak phosphorylation levels typically occurring 5–10 minutes after maximal resistance exercise, which precisely aligns with the 9-minute peak performance observed in our HBS cohort. Second, central nervous system excitability undergoes dynamic modulation following maximal effort: initial post-exercise inhibition of spinal α-motor neurons and cortical motor areas gradually resolves, with peak neural drive typically returning 8–12 minutes after exercise cessation (Tillin and Bishop, 2009). Third, transient alterations in muscle-tendon unit mechanics, including temporary reductions in tendon stiffness and shifts in the muscle length-tension relationship, require several minutes to return to optimal contractile conditions. Furthermore. A recent meta-analysis specifically examining back squat-induced PAPE found that the optimal rest interval ranges from 4–9 minutes (Chen et al., 2023), which is in excellent agreement with our observation of peak performance at 9 minutes. However, earlier meta-analyses have reached divergent conclusions. Gouvea et al. identified 8–12 minutes as the optimal window (R Core Team, 2025), while Dobbs et al. reported superior jumping performance at 3–7 minutes (Dobbs et al., 2019). These discrepancies are almost certainly attributable to key methodological differences between studies, including variations in participant training experience (elite athletes vs. recreational exercisers), the duration and intensity of the preceding general warm-up, and the temporal resolution of testing time points.

In sharp contrast to the constant external load of the barbell, the MEB protocol capitalizes on the mechanical properties of accommodating resistance. This distinct loading profile ensures that the resistance load decreases during the eccentric phase and at biomechanically weaker joint angles, thereby matching the human strength curve more efficiently. We propose that this variable loading profile likely imposed lower mechanical stress and may have resulted in reduced metabolic byproduct accumulation compared to the systemic demands of the heavy barbell squat, though this mechanistic inference is speculative as direct metabolic markers were not measured in this study. As demonstrated by Sabag et al. loads exceeding 85% 1RM lead to disproportionate fatigue accumulation (Sabag et al., 2018), which often results in the “fatigue-masking effect” dominating and abrogating the net benefit of PAPE. In contrast, the MEB protocol appears to strike a more favorable balance between inducing sufficient potentiation and minimizing fatigue accumulation. Consequently, this favorable fatigue-management profile may have allowed the underlying potentiation effect to outweigh the dissipating fatigue much earlier, specifically within 3 minutes post-activation. This observation of rapid recovery and potentiation trend is consistent with recent findings by Shi and Ye (Shi et al., 2023) and Katushabe et al. (Katushabe and Kramer, 2020), who have similarly suggested that variable resistance training offers a superior fatigue-management profile for the efficient induction of PAPE.

The analysis of physiological kinetic data provides further mechanistic insight into the observed performance changes. Although our statistical analysis did not detect significant main effects for changes in leg stiffness, the maintenance of stable stiffness levels throughout the protocol is a positive indicator regarding the neuromuscular state. It suggests that the MEB protocol did not induce the type of negative neuromuscular fatigue that typically impairs the efficiency of the Stretch-Shortening Cycle (SSC) (Struzik and Zawadzki, 2013). This is critical because maintaining higher leg stiffness allows for the optimal storage and subsequent reutilization of elastic strain energy within the musculotendinous unit, which is recognized as a key underlying mechanism contributing to the PAPE phenomenon (Cormie et al., 2010).

From a practical coaching standpoint, the accurate identification of the optimal recovery time window is crucial for the successful implementation of warm-up strategies. Our effect size analysis indicates that the MEB protocol offers a flexible practical performance window ranging from 3 to 15 minutes, with the most pronounced practical effects observed between 3 and 6 minutes. This temporal flexibility is advantageous in unpredictable competition settings, such as player substitutions in team sports or call-room delays in track and field events, where warm-up time is often severely constrained. Conversely, the HBS protocol requires a recovery period of at least 9 minutes to achieve practical performance improvements, a requirement that may be logistically challenging to implement immediately prior to competition. Therefore, we recommend that strength and conditioning coaches prioritize multi-dimensional elastic bands for acute “pre-competition” activation to ensure immediate readiness, while reserving heavy squats for “complex training” sessions during the preparatory phase where longer rest periods are feasible and can be afforded.

## 5 Study limitations

Several limitations should be noted when interpreting these findings. First, the relatively small sample size (n = 16 male collegiate athletes) may have limited the statistical power of the study. Therefore, the observed trends and effect size findings should be considered preliminary and require validation in larger sample studies. Additionally, the results may not be generalizable to female athletes (who may exhibit different fatigue resistance) or elite-level populations with higher baseline strength levels. Second, no electromyography (EMG) measurements were performed to assess neuromuscular activation patterns. This prevents us from elucidating the underlying physiological mechanisms responsible for the observed performance trends. Future studies should incorporate surface EMG to quantify muscle activation amplitude and timing during the warm-up and subsequent jump tests. Third, the biomechanical assessment was limited to basic jump performance parameters (height, peak power, flight time, and leg stiffness). Direct measurements of tendon stiffness and detailed force-plate kinetics were not obtained. These measurements would provide deeper insights into how different warm-up protocols modify the mechanical properties of the musculoskeletal system and influence explosive movement mechanics. Fourth, potential learning and session order effects cannot be completely ruled out. The study employed a fixed-sequence crossover design to minimize practice effects by placing the familiar standard warm-up condition first. While a one-week washout period was implemented and no significant residual effects were expected from acute warm-up interventions, this design inherently prevents the statistical separation of order effects from true condition effects. Future studies could adopt a randomized crossover design to systematically balance and control for potential order effects. Fifth, we did not measure metabolic markers (e.g., blood lactate) directly, relying instead on performance decrements to infer fatigue. Direct physiological measurements would strengthen the “fitness-fatigue” argument and help clarify the temporal relationship between metabolic recovery and performance restoration following different activation protocols. Sixth, a discrepancy in intensity standardization exists between protocols. The HBS condition was prescribed using an external mechanical load (%1RM), whereas the MEB intensity was regulated via internal physiological response (heart rate). While heart rate monitoring is common in field-based warm-ups, it may not perfectly reflect the mechanical tension applied to the musculature compared to weight-based prescriptions. Consequently, the observed differences in the time-course of PAPE may partly reflect the distinct physiological demands of the load regulation strategies rather than the resistance type alone. This methodological mismatch limits the direct causal inference of the observed effects between the two resistance modalities. Future research should investigate the dose-response relationship of elastic band tension (e.g., comparing different band stiffness levels) and explore its effects on other sport-specific tasks, such as on-field sprint performance or agility tasks, to broaden the applicability of MEB as a universal warm-up tool.

## 6 Conclusion

In conclusion, the present study demonstrates that the Multi-dimensional Elastic Band (MEB) protocol offers a time-efficient alternative for inducing Post-activation Performance Enhancement (PAPE) in lower limb explosive tasks compared to traditional heavy resistance loading. The MEB protocol elicits a rapid onset of practical performance improvement (within 3 minutes) and maintains this effect for up to 15 minutes, while the traditional heavy barbell squat protocol requires >9 minutes of recovery to achieve comparable practical benefits, likely due to the greater acute neuromuscular fatigue associated with heavy resistance loading.

From a practical perspective, these findings suggest that strength and conditioning coaches should prioritize multi-dimensional elastic band exercises during pre-competition warm-ups or time-constrained substitution scenarios, where immediate readiness is paramount. Conversely, traditional heavy back squats may be better reserved for complex training sessions during the preparatory phase, where longer rest intervals can be afforded to dissipate fatigue. Future research should explore the chronic adaptations of integrating this activation method into long-term periodization models.

## Data Availability

The raw data supporting the conclusions of this article will be made available by the authors, without undue reservation.
